# Associations of prenatal exposure to per- and polyfluoroalkyl substances (PFAS) and childhood vaccine-induced immunity

**DOI:** 10.1186/s12940-026-01322-7

**Published:** 2026-07-15

**Authors:** Anna Smith, Pi-I D. Lin, Sheryl L. Rifas-Shiman, Diane R. Gold, Marie-France Hivert, Emily Oken, Andres Cardenas

**Affiliations:** 1 Department of Epidemiology and Population Health, Stanford University, Stanford, CA USA; 2 Division of Chronic Disease Research Across the Lifecourse, Department of Population Medicine, Harvard Medical School, Harvard Pilgrim Health Care Institute, Boston, MA USA; 3 Channing Division of Network Medicine, Department of Medicine, Harvard Medical School and Brigham and Women’s Hospital, Boston, MA USA; 4 Department of Environmental Health, Harvard T.H. Chan School of Public Health, Boston, MA USA; 5 Diabetes Unit, Massachusetts General Hospital, Boston, MA USA; 6 Department of Pediatrics, Stanford University, Stanford, CA USA; 7 Department of Epidemiology and Population Health, Stanford University, 1701 Page Mill Rd, Palo Alto, CA 94304 USA

**Keywords:** Antibodies, Children’s health, Epidemiology, Immunity, Maternal and fetal exposure/health, Mixtures, PFAS, Population based studies

## Abstract

**Background:**

Plasma per- and polyfluoroalkyl substances (PFAS) are associated with immune dysfunction, including childhood antibody response. We evaluated whether first trimester maternal plasma PFAS concentrations are associated with mid-childhood MMR (measles, mumps, rubella) and DTaP (diphtheria, tetanus, pertussis) antibody titers.

**Methods:**

We measured six PFAS (EtFOSSA, MeFOSSA, PFHxS, PFNA, PFOA, PFOS) in first trimester plasma from participants in the longitudinal Project Viva cohort, recruited 1999–2002 in eastern Massachusetts. We measured mid-childhood plasma MMR and DTaP antibody titers. We restricted the analytical sample to children fully vaccinated, according to 2007 United States Center for Disease Control guidelines and used covariate-adjusted quantile g-computation and regression analyses to estimate associations (*n* = 333–416).

**Results:**

Median [interquartile range (IQR)] age at antibody titer blood draw was 7.7 years (7.4, 8.4), and children received their most recent MMR or DTaP dose at median (IQR) 4.3 years (4.1, 5.0). The PFAS mixture was associated with lower antibody titers, except for measles, although none of the associations reached statistical significance. A one unit (ng/mL) increase in EtFOSSA was associated with higher measles antibody titers [β = 0.02, 95% confidence interval (CI) = 0.01, 0.04 antibody (Ab) index]. A one ng/mL increase in MeFOSSA was associated with − 6.8% (95% CI: -11.9, -1.3) lower pertussis antibody titers. There was evidence of effect modification by infant sex for prenatal PFOS and childhood pertussis antibody titers, with higher prenatal PFOS associated with lower antibody titers in females and higher in males, although neither association reached statistical significance.

**Conclusions:**

While the prenatal PFAS mixture was not significantly associated with childhood antibody titers in our cohort, we observed individual associations between EtFOSSA and higher measles antibody titers and MeFOSSA and lower pertussis antibody titers. Overall, we found limited evidence of associations between first trimester plasma PFAS and mid-childhood antibody titers in our cohort.

**Trial registration:**

Clinical trial number: not applicable.

**Supplementary Information:**

The online version contains supplementary material available at https://doi.org/10.1186/s12940-026-01322-7.

## Introduction

Environmental exposures influence human health, including immune function [1, 2]. Most of the innate immune system is established *in utero* and acts as the first line of defense from pathogens [3, 4], initiating the development of trained immunity and providing nonspecific protection against broad pathogen types [4]. Adaptive immune responses from T and B cells develop soon after, providing long-term, pathogen-specific protection, and the rate at which a neonate shifts from an early adaptive immune system toward mature immune memory may determine vaccination efficacy [4]. This period of fetal development is sensitive to exogenous exposures, some of which may have lasting effects on the developing immune system [3]. For example, per- and polyfluoroalkyl substances (PFAS) are a family of ubiquitous, stable, synthetic chemicals, and human exposure is associated with immune dysfunction [5]. Humans are exposed through drinking water, food items, indoor dust, air, and household products [6, 7], and PFAS are widely detected in pregnant persons [8]. PFAS accumulate in the placenta and cross the placental barrier, exposing the developing embryo [8]. PFAS are also detected in umbilical cord blood, breast milk, and plasma of breastfed toddlers, reflecting ongoing perinatal exposure [1, 9]. PFAS induce immunosuppression and alter cytokine levels and cell signaling involved in immune function [1, 10]. PFAS have key characteristics of immunotoxic agents [11], which include modulating cell populations involved in antibody responses, interfering with the processing and presentation of antigens, and altering cell death processes through oxidative stress [1].

In 2016, the United States National Toxicology Program (NTP) concluded that two widespread legacy PFAS, perfluorooctanoic acid (PFOA) and perfluorooctanesulfonic acid (PFOS), are immune hazards in humans based on strong evidence that they are associated with lower antibody responses from vaccination in experimental animals and moderate evidence in humans [12]. In 2021, the United States Agency for Toxic Substances and Disease Registry added perfluorohexane sulfonic acid (PFHxS) and perfluorodecanoic acid (PFDA) to the list of PFAS associated with reduced vaccine-induced antibody responses in humans in epidemiological studies [9]. Recent epidemiological studies have found associations specifically between prenatal and early and mid-childhood PFAS and lower childhood vaccine-induced antibody titers, especially for diphtheria and tetanus [13–21]. More data is available for associations of childhood PFAS with vaccine titers than for associations of prenatal PFAS with vaccine titers, and studies have not previously focused on exposure during the first trimester of pregnancy.

Routine childhood vaccinations present an opportunity to study PFAS exposure during development and immunity. Although highly effective, a small percentage of children will have primary (lack of seroconversion) or secondary (waning immunity) vaccine failure [22], leaving them vulnerable to preventable illness in settings of low vaccine uptake [23–25]. We leveraged a prospective pre-birth cohort to examine associations of prenatal PFAS with childhood MMR (measles, mumps, rubella) and DTaP (diphtheria, tetanus, pertussis) antibody titers. We hypothesized that the PFAS mixture and individual PFAS would be associated with lower measles, mumps, rubella, pertussis, diphtheria, and tetanus antibody titers.

## Methods

### Study population

We recruited pregnant persons from eastern Massachusetts, USA, to the prospective pre-birth cohort study, Project Viva, between 1999 and 2002, during their first prenatal visit (median = 10 weeks gestation) [26]. Eligibility included English fluency, singleton pregnancy, gestational age less than twenty two weeks, and plans to remain in the study area during the gestational period.

Of 2128 mother-child pairs, 28 participants had repeat pregnancies enrolled in the cohort, and we used data from the first completed pregnancy (*n* = 2100) of which, 1627 had maternal first trimester PFAS measurements. We measured at least one antibody titer outcome in 523 of the children at mid-childhood, and 486 of these children had medical record data available. We restricted the sample size to children who had evidence of having been fully vaccinated with the MMR [or measles, mumps, rubella, and varicella (MMRV) vaccine] and DTaP vaccine series. Of the 486 participants, 424 children had two confirmed doses of the MMR vaccine and 414 children had five confirmed doses of the DTaP vaccine. We also restricted the sample size to fully vaccinated participants who received their vaccinations according to the United States Centers for Disease Control and Prevention (CDC) 2007 vaccination schedule, as described in our prior study [21, 27], reducing the sample sizes to 423 children and 412 children for the MMR and DTaP analytical samples. Finally, we excluded children who received their last vaccine dose less than two weeks prior to antibody titer blood draw. After these exclusion criteria, 416 participants remained in our study population for the MMR antibody titer analyses and 405 participants for the DTaP antibody titer analyses (Figure S1).

We followed STROBE reporting guideline for cohort studies. Participants provided written informed consent at recruitment and all postnatal follow-up visits, and children provided verbal assent at mid-childhood. The Institutional Review Board of Harvard Pilgrim Health Care reviewed and approved all study protocols.

### Exposure assessment

Prior studies described methods for measuring plasma PFAS concentrations [10, 28]. Upon recruitment, we collected a blood sample from participants. The Division of Laboratory Sciences at the Centers for Disease Control and Prevention (CDC) (Atlanta, Georgia, USA) measured plasma concentrations of perfluorooctane sulfonate (PFOS), perfluorooctanoate (PFOA), perfluorohexane sulfonate (PFHxS), perfluorononanoate (PFNA), 2-(N-ethyl-perfluorooctane sulfonamide) acetate (EtFOSAA), and 2-(N-methyl-perfluorooctane sulfonamide) acetate (MeFOSAA), following standard protocols [29]. The limit of detection (LOD) and detection frequency (> 99%) for each PFAS [28] are shown in Table S1. We assigned concentrations below the LOD the value LOD/(square root of 2) and report results as plasma PFAS concentrations (ng/mL).

### Vaccine titers

We describe methods for analyzing vaccine-induced antibody titers in our previous study [21]. Briefly, we stored biobanked childhood heparin plasma at -80 °C and transported on dry ice to Boston Children’s Hospital. We thawed samples at room temperature. We vortexed and centrifuged samples at 3000 rpm prior to manual testing. We analyzed MMR and DTaP antibody titers in duplicate following assay insert instructions. Assays included the Measles IgG ELISA Kit (Abnova, Taipei City, Taiwan), Mumps IgG ELISA Kit (Abnova, Taipei City, Taiwan), Rubella virus IgG ELISA Kit (Abnova, Taipei City, Taiwan), Bordetella pertussis Toxin IgG ELISA Kit (Abnova, Taipei City, Taiwan), Human Anti-Diptheria Toxoid IgG ELISA Assay (XpressBio, Frederick, MD, USA), and the Human Anti-Tetanus Toxoid IgG ELISA Assay (XpressBio, Frederick, MD, USA). There were fewer participants included in some of the antibody outcome measurement outcomes, such as tetanus, due to low plasma concentrations, and we prioritized measuring antibody titers that were studied less in prior literature, such as measles and mumps. Antibody titers for each assay were reported continuously and categorically, and a summary of the assays and seroprotective cutoffs are detailed in our prior manuscript [21].

### Covariate assessment

At study enrollment, participants self-reported demographic characteristics during an interview and questionnaire. We chose covariates based on our directed acyclic graph (DAG) (Figure S2) for the relationship between first trimester PFAS and mid-childhood measles, mumps, rubella, pertussis, diphtheria, and tetanus. We included assigned child sex at birth, abstracted from delivery records, as a covariate in main models. We also conducted sensitivity analyses examining assigned child sex at birth as an effect modifier. There is evidence from prior studies that time since immunization has a strong influence on antibody levels [22, 30], and so we adjusted for time since most recent immunization (dose number two for MMR analyses and dose number five for DTaP analyses). There is also evidence that parity may influence both prenatal PFAS levels (through potential breastfeeding in prior pregnancies) [31] and mid-childhood antibody titers (due to parity-associated variations in immune cell composition of human milk) [32, 33], and so we adjusted for this variable as well.

Since we restricted our study sample to fully vaccinated participants, we did not adjust for maternal age at enrollment, education (proxy for socioeconomic status), and race and ethnicity, which are associated with prenatal PFAS and number of vaccine doses in prior studies [8, 34–36]. We also considered breastfeeding as a potential confounder due to its known impact on PFAS exposure and vaccine-induced immunity [8, 37, 38], however, we did not include this variable since it was a post-exposure variable and could be on the causal pathway (Figure S2), since PFAS are linked with insufficient mammary gland development and milk production during lactation in animal studies, as well as reduced breastfeeding duration in epidemiological studies [39].

### Statistical analysis

We used adjusted quantile g-computation with linear regression models [40] to estimate the overall association between the PFAS mixture and each antibody titer outcome. Quantile g-computation is a parametric, generalized linear model-based implementation of g-computation [40], that estimates parameters of a marginal structure model that characterize the change in expected antibody titer outcomes given a join intervention of all PFAS, conditional on confounders. The estimate is interpreted as associations in each outcome for a one quartile simultaneous increase in all PFAS in the mixture, referred to as the PFAS mixture exposure-response [40].

We conducted linear regression analyses to estimate associations between individual PFAS with continuous antibody titers, adjusting for covariates. The MMR outcomes are interpreted as the estimated change in antibody titer outcome for a one unit (ng/mL) increase in PFAS concentration. For the DTaP models, we log-transformed the titer outcomes to meet model assumptions of normally distributed residuals, and estimates and confidence intervals (CIs) were converted using the formulas (e^β^-1)*100 and (e^95% CI upper or lower bound^-1)*100 [41] and interpreted as a the relative percent change (95% CI) in the median of antibody titer associated with one ng/mL change in PFAS concentration. We converted 95% CIs to evaluate magnitude of associations and statistical significance [41]. In addition, we checked for effect modification between each PFAS and child sex, using linear regression models with a multiplicative term. When an interaction term was statistically significant at α < 0.05, we examined associations between the individual PFAS and outcomes, stratified by child sex. As described in our prior study [21], we considered examining outcomes categorically, but did not for several reasons: First, in the general population, two age-appropriate MMR vaccine doses is considered sufficient protection [42, 43]. Second, antibody titers are not the only mechanisms of immunity post-vaccine [42–44]. Third, protective thresholds vary by assay [45], except for diphtheria and tetanus in which all participants were considered above the threshold [46]. Our study focused on examining how PFAS may impact immunity, and not on whether antibody titer levels are sufficient to prevent disease.

## Results

### Baseline characteristics

We report baseline characteristics for our MMR and DTaP analytical samples in Table 1. In our MMR analytical sample (*n* = 416), median [interquartile range (IQR)] maternal age of participants was 32.5 years (28.6, 35.6). Most maternal participants self-identified their race as White (65.1%), had completed a college education (63.9%), and were parous (57.9%). There were more male (54.3%) than female (45.7%) children (Table 1). Distributions of demographic characteristics were nearly identical in the DTaP analytical sample (*n* = 405) (Table 1); 383 participants were included in both samples. Median (IQR) child age at blood draw was 7.7 years (7.4, 8.4), and the median (IQR) age that the children received their most recent MMR (dose number 2) and DTaP (dose number 5) vaccinations was 4.3 years (4.1, 5.0) (Table 1).

**Table 1:** Characteristics of mother-child pairs from the Project Viva cohort with prenatal PFAS measurements, at least one antibody titer outcome, and fully vaccinated on schedule with the MMR or DTaP vaccine

Demographic Characteristics		Completed MMR series	Completed DTaP series
		*n* (%) or median (IQR)	*n* (%) or median (IQR)
Overall		416	405
Maternal age at enrollment (years)		32.5 (28.6, 35.6)	32.6 (28.6, 35.9)
Maternal race or ethnicity			
	Asian	14 (3.4)	14 (3.5)
	Black	80 (19.2)	75 (18.5)
	Hispanic	27 (6.5)	27 (6.7)
	White	271 (65.1)	266 (65.7)
	More than one^a^	21 (5.0)	22 (5.4)
	No self-report	3 (0.7)	1 (0.2)
Maternal education			
	College graduate	266 (63.9)	259 (64.0)
	Not a college graduate	147 (35.3)	145 (35.8)
	No self-report	3 (0.7)	1 (0.2)
Parity			
	Nulliparous	175 (42.1)	173 (42.7)
	One or more	241 (57.9)	232 (57.3)
Child assigned sex at birth			
	Female	190 (45.7)	191 (47.2)
	Male	226 (54.3)	214 (52.8)
Child age titer blood draw (years)		7.7 (7.4, 8.4)	7.7 (7.4, 8.3)
Child age last vaccine dose (years)		4.3 (4.1, 5.0)	4.3 (4.1, 5.0)
Child time since last vaccine (years)^b^		3.3 (2.7, 3.9)	3.3 (2.7, 3.9)

Abbreviations: *%* percent, *IQR* Interquartile range, *PFAS* per- and polyfluoroalkyl substances

^a^Includes American Indian or Alaskan Native, Asian or Pacific Islander, Black or African American, Hispanic or Latina, White or Caucasian, and Other, Specify, as written in the self-report survey

^b^Time since last vaccine at the time of titer blood draw for child

### PFAS correlations and distributions

We present PFAS distributions for the analytical sample (Table 2) and Spearman correlation coefficients among PFAS in both the MMR and DTaP analytical samples (Figure S3). The correlations were nearly identical in both samples, and PFAS were moderately correlated, with PFOS and PFOA having the highest correlation (*r* = 0.72).

**Table 2:** Distributions of per- and polyfluoroalkyl substances (PFAS) in first trimester blood for participants with all PFAS measurements, at least one antibody titer outcome, and a confirmed completed MMR vaccine series^a^ (*n* = 416)

PFAS^b^	Median (IQR)	Minimum	Maximum	% > LOD	LOD
EtFOSAA (ng/mL)	1.2 (0.7, 1.8)	0.07	18.0	99	0.1
MeFOSAA (ng/mL)	2.0 (1.3, 3.2)	0.1	30.0	100	0.1
PFHxS (ng/mL)	2.4 (1.6, 3.7)	0.07	43.0	99	0.1
PFNA (ng/mL)	0.6 (0.5, 0.9)	0.07	3.4	99	0.1
PFOA (ng/mL)	5.5 (3.8, 7.5)	0.9	22.0	100	0.1
PFOS (ng/mL)	24.6 (18.1, 33.8)	4.6	170.0	100	0.2

Abbreviations: *EtFOSAA* 2-(N-ethyl-perfluorooctane sulfonamide) acetate, *IQR* Interquartile range, *LOD* Limit of detection, *MeFOSAA* 2-(N-Methyl-perfluorooctane sulfonamido) acetic acid, *PFAS* Per- and polyfluoroalkyl substances, *PFHxS* Perfluorohexane sulfonate, *PFNA* Perfluorononanoic acid, *PFOA* Perfluorooctanoic acid, *PFOS* Perfluorooctane sulfonate

^a^PFAS Distributions for participants with at least one antibody titer outcome and a complete DTaP vaccine series were the same (*n* = 405)

^b^PFAS concentrations are presented per mL of plasma

### Mid-childhood antibody titer correlations and distributions

**Fig. 1:**
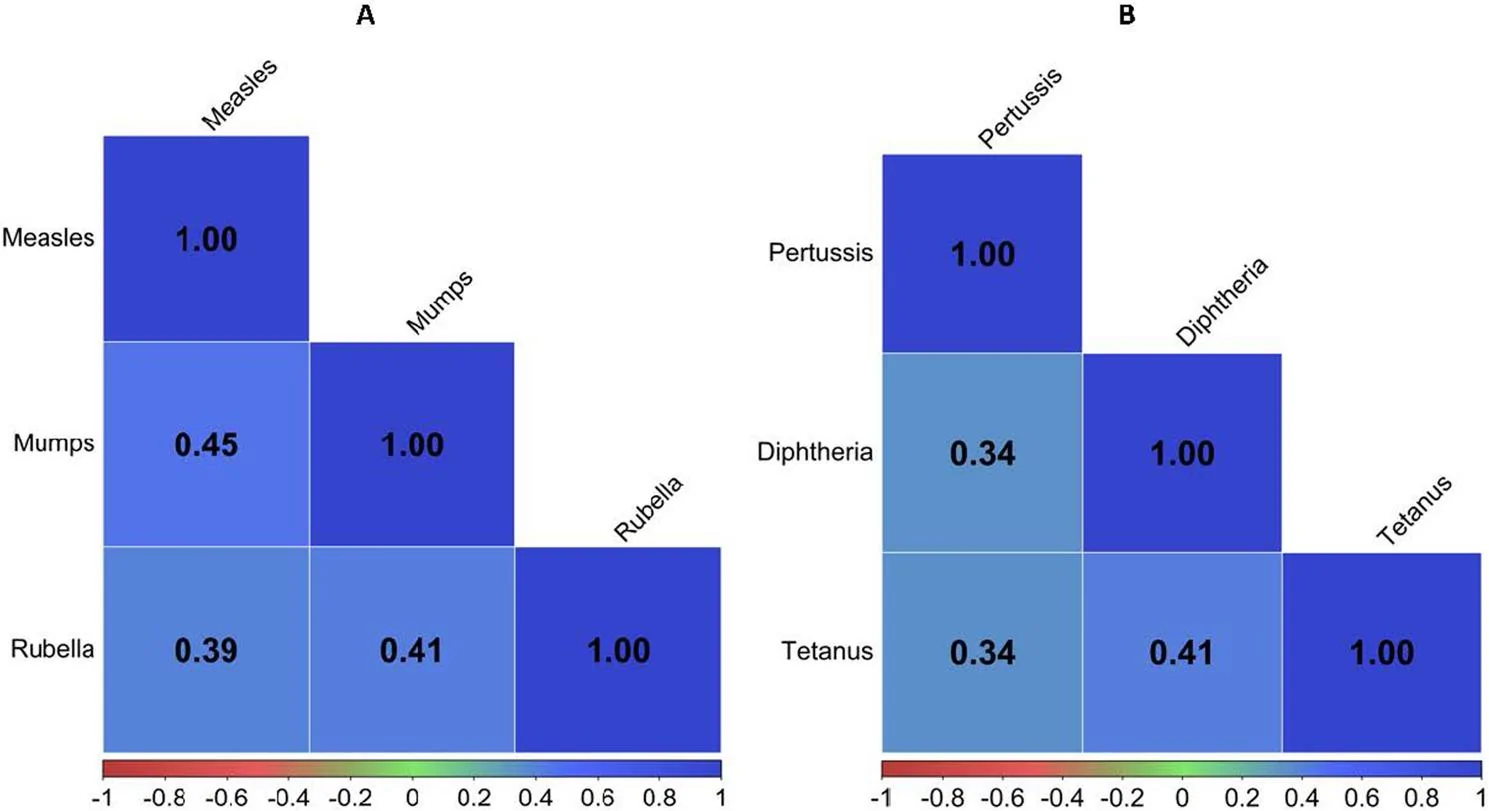
Spearman correlation coefficients among continuous antibody titer outcomes in participants with at least one antibody titer outcome, and (**A**) a complete MMR vaccine series (n=416) or (**B**) a complete DTaP vaccine series (n=405)

We present Spearman correlation coefficients among measles, mumps and rubella antibody titers, as well as pertussis, diphtheria, and tetanus antibody titers (Fig. 1), which were all positively and moderately correlated. We present Spearman correlation coefficients among all antibody titers in Figure S4, and MMR antibody titers have moderate to low correlation with DTaP antibody titers. We observed the strongest correlation between measles and mumps (*r* = 0.45), followed closely by mumps and rubella (*r* = 0.41) and diphtheria and tetanus (*r* = 0.41). For reference, we present continuous antibody titer distributions for participants in Table 3 and categorical distributions based on seroprotective threshold assay cutoffs, as described in our prior manuscript [21] in table s1.

**Table 3:** Distributions of antibody titers in mid-childhood blood for participants with prenatal PFAS measurements, at least one antibody titer outcome, and fully vaccinated on schedule with the MMR or DTaP vaccine

Antibody Titers	Total *n*		Median (IQR) (Ab index or IU/mL)^a^	Minimum	Maximum
Measles		416	1.11 (0.88, 1.30)	0.20	2.00
Mumps		416	2.85 (2.15, 3.51)	0.32	5.60
Rubella		402	32.93 (20.94, 46.81)	3.00	81.00
Pertussis		396	6.18 (2.97, 12.81)	-0.09	2200.00
Diphtheria		388	0.51 (0.31, 0.82)	0.07	4.00
Tetanus		333	0.99 (0.43, 2.07)	0.03	39.00

Abbreviations: IQR, interquartile range; *n*, sample size; PFAS, per- and polyfluoroalkyl substances

^a^Measles and mumps units are Ab index; rubella, pertussis, diphtheria, and tetanus units are IU/mL

### Associations of PFAS with antibody titers

The PFAS mixture in maternal prenatal plasma was associated with higher child measles titers and lower mumps, rubella, pertussis, diphtheria, and tetanus antibody titers, although none of these associations reached statistical significance (Table 4). In individual analyses, a one unit (ng/mL) higher prenatal EtFOSSA was associated with higher measles antibody titers [β = 0.02, 95% CI = 0.01, 0.04 antibody (Ab) index)], while we did not observe statistically significant associations between any of the PFAS and the mumps and rubella antibody titers (Table 5). A one ng/mL increment in MeFOSSA concentration was associated with − 6.8% (95% CI: -11.8, -1.3) lower pertussis antibody titers, while we did not observe statistically significant associations between any of the PFAS and diphtheria and tetanus antibody titers (Table 6).

**Table 4:** Quantile g-computation exposure-response estimates (95% bootstrap confidence intervals) for associations of the prenatal PFAS mixture (ng/mL) and measles, mumps (Ab index), rubella, pertussis, diphtheria, and tetanus (IU/mL) antibody titers

Mixture	Antibody Titer^a^	β (95% bootstrap CI)^b, c^
PFAS (EtFOSSA, MeFOSSA, PFHxS, PFNA, PFOA, PFOS)	Measles (*n* = 416)	0.02 (-0.02, 0.06)
	Mumps (*n* = 416)	-0.02 (-0.16, 0.11)
	Rubella (*n* = 402)	-1.2 (-3.4, 1.1)
	Pertussis (*n* = 396)	-0.12 (-0.3, 0.04)
	Diphtheria (*n* = 388)	-0.05 (-0.1, 0.05)
	Tetanus (*n* = 333)	0.00 (-0.2, 0.2)

Abbreviations: *Ab* Antibody, *CI* Confidence interval, *EtFOSAA* 2-(N-ethyl-perfluorooctane sulfonamide) acetate, *MeFOSAA* 2-(N-Methyl-perfluorooctane sulfonamido) acetic acid, *PFAS* Per- and polyfluoroalkyl substances, *PFHxS* Perfluorohexane sulfonate, *PFNA* Perfluorononanoic acid, *PFOA* Perfluorooctanoic acid, *PFOS* Perfluorooctane sulfonate, *n* sample size

^a^Sample sizes are different due to insufficient plasma to measure all antibody titers in all the participants

^b^For measles, mumps, and rubella antibody titer models, estimates interpreted as the difference in each outcome for one-quartile increase in all PFAS, conditional on the covariates assigned sex at birth, time since last vaccination, and parity

^c^For pertussis, diphtheria, and tetanus antibody titers models, estimates interpreted as the difference in each log-outcome for one-quartile increase in all PFAS, conditional on the covariates assigned child sex at birth, time since last vaccination, and parity

**Table 5:** Associations of individual prenatal blood PFAS (ng/mL) and measles (*n* = 416), mumps (*n* = 416) (Ab index), and rubella (*n* = 402) (IU/mL) antibody titers

PFAS	Measles β (95% CI)^a^	Mumps β (95% CI)^a^	Rubella β (95% CI)^a^
EtFOSSA	0.02 (0.01, 0.04)	0.01 (-0.05, 0.07)	-0.6 (-1.6, 0.4)
MeFOSSA	-0.01 (-0.02, 0.01)	-0.02 (-0.07, 0.02)	-0.3 (-1.1, 0.4)
PFHxS	-0.01 (-0.01, 0.002)	-0.01 (-0.04, 0.01)	-0.2 (-0.6, 0.2)
PFNA	-0.04 (-0.12, 0.05)	-0.01 (-0.3, 0.3)	0.5 (-4.1, 5.1)
PFOA	0.004 (-0.01, 0.02)	-0.005 (-0.04, 0.03)	0.04 (-0.5, 0.6)
PFOS	0.002 (-0.0003, 0.004)	0.00 (-0.01, 0.01)	0.01 (-0.1, 0.1)

Abbreviations: *Ab* Antibody, *CI* Confidence interval, *EtFOSAA* 2-(N-ethyl-perfluorooctane sulfonamide) acetate, *MeFOSAA* 2-(N-Methyl-perfluorooctane sulfonamido) acetic acid, *PFAS* Per- and polyfluoroalkyl substances, *PFHxS* Perfluorohexane sulfonate, *PFNA* Perfluorononanoic acid, *PFOA* Perfluorooctanoic acid, *PFOS* Perfluorooctane sulfonate, *n*, sample size

^a^Estimates interpreted as the estimated change in antibody titer outcome for a one unit (ng/mL) increase in PFAS concentration. Models adjusted for assigned child sex at birth, time since last vaccination, and parity

**Table 6:** Associations of individual prenatal blood PFAS (ng/mL) and pertussis (*n* = 396), diphtheria (*n* = 388), and tetanus (*n* = 333) antibody titers (IU/mL)

PFAS	Pertussis %Δ (95% CI)^a^	Diphtheria %Δ (95% CI)^a^	Tetanus %Δ (95% CI)^a^
EtFOSSA	-6.2 (-13.4, 1.6)	-1.4 (-5.6, 2.9)	2.9 (-4.4, 10.8)
MeFOSSA	-6.8 (-11.8, -1.3)	-0.4 (-3.4, 2.8)	3.8 (-1.6, 9.5)
PFHxS	1.9 (-1.1, 5.0)	0.1 (-1.5, 1.8)	0.4 (-3.0, 3.8)
PFNA	-14.5 (-41.3, 24.6)	-8.9 (-25.9, 11.9)	0.7 (-29.3, 43.4)
PFOA	-1.7 (-5.9, 2.7)	1.2 (-1.1, 3.7)	2.0 (-2.1, 6.3)
PFOS	0.1 (-0.7, 0.9)^**†**^	-0.05 (-0.5, 0.4)	0.2 (-0.6, 0.9)

Abbreviations: *%Δ* percent change, *CI* Confidence interval, *EtFOSAA* 2-(N-ethyl-perfluorooctane sulfonamide) acetate, *MeFOSAA* 2-(N-Methyl-perfluorooctane sulfonamido) acetic acid, *PFAS* Per- and polyfluoroalkyl substances, *PFHxS* Perfluorohexane sulfonate, *PFNA* Perfluorononanoic acid, *PFOA* Perfluorooctanoic acid, *PFOS* Perfluorooctane sulfonate, *n* sample size

^a^We log-transformed the antibody titer outcomes and estimates and confidence intervals were converted using the formulas (e^β^-1)*100 and (e^95% CI upper or lower bound^-1)*100 and interpreted as the relative change in median antibody titer [(%Δ (95% CI)] when adding one unit of PFAS concentration. Models adjusted for assigned child sex at birth, time since last vaccination, and parity

^**†**^Sex-interaction *p*-value = 0.007

There was evidence of effect modification by infant sex in prenatal PFOS and childhood pertussis models (p-value interaction = 0.007). In stratified models, PFOS was associated with lower pertussis antibody titers in females (%Δ=-0.9, 95% CI: -2.0, 0.2) and higher in males (%Δ = 1.0, 95% CI: -0.1, 2.1), but neither association reached statistical significance (Table S2).

## Discussion

In our longitudinal cohort study of children fully vaccinated on schedule with the MMR and DTaP vaccines, we found few statistically significant associations between prenatal first trimester PFAS and mid-childhood antibody titers. While we did not observe associations between the PFAS mixture and any of the antibody titers, we did observe an association between higher EtFOSSA and higher measles antibody titers, which was contrary to our a priori hypothesis. Consistent with our hypothesis, we observed an association between MeFOSSA and lower pertussis antibody titers. We also observed sex-specific associations between PFOS and pertussis antibody titers, although none of the stratified estimates were statistically significant.

A similar study in participants from the Healthy Start Cohort, recruited 2009–2014 during pregnancy (< 24 weeks gestation), in Colorado, United States, evaluated associations between mid- to late pregnancy blood PFAS and childhood (4–8 years) measles, mumps, rubella, and varicella antibody titers (*n* = 145) [13]. The Healthy Start Cohort found an association between prenatal PFNA and a higher odds of having non–high-censored (or “low”) measles antibody titers, as well as prenatal PFOA and a higher odds of low mumps antibody titers, while they did not observe significant associations between any of the PFAS and rubella or varicella titers [13]. Similar to our cohort, the Healthy Start Cohort observed sex-specific associations, and they found that females in particular had a higher odds of a low mumps antibody titers in association with higher PFNA and PFDA, and that PFOA, PFNA, and PFDA were each associated with lower rubella and varicella antibody titers in females and higher in males [13]. Also similar to our cohort, Healthy Start Cohort did not identify significant associations between the PFAS mixture (PFDA, PFHxS, PFNA, PFOA, PFOS) and antibody titers, although they harnessed a different mixture [13]. Differences in study findings could be PFAS exposure timing, since the production of certain PFAS, such as PFOA and PFOS, declined in the USA, along with blood PFAS [47], as well as differences in analysis methods. For example, the Healthy Start Cohort used categorical measles and mumps antibody titer cutoffs, defining the cutoff as titers above or below the assay reporting limit, whereas we used linear antibody titer classifications in the Project Viva study.

Another recent study evaluated associations between eleven PFAS measured in mid-pregnancy serum (24–26 weeks gestation), in 2011, from participants in Hamburg, Germany and childhood (age five years) measles, mumps, rubella, diphtheria, and tetanus antibody titers (*n* = 152) [32]. In that study, prenatal PFHxS was associated with lower diphtheria and tetanus antibody titers, PFHpS was associated with lower tetanus antibody titers, and PFUnDA was associated with lower rubella antibody titers. The study did not find statistically significant associations between any of the PFAS and measles and mumps antibody titers. Similar to our Project Viva cohort and the Healthy Start cohort, the study in Germany found evidence of effect modification by child sex and found several associations between specific PFAS and diphtheria and rubella antibody titers in female children only, as well as one inverse association between PFHxS and mumps antibody titers in male children only [32]. Similar to the Healthy Start cohort, differences in study findings could be due to differences in year of pregnancy and PFAS prevalence. In addition, the German cohort harnessed a younger cohort of children, so the time between vaccination and titer measurement might have been shorter.

Contrary to our findings, a study examining late pregnancy PFAS (PFOS, PFOA, PFXsS, PFNA, PFDA) and childhood antibodies in participants from the Faroe Islands (*n* = 532) found that PFOA was associated with lower mid-childhood (age five years) diphtheria antibodies [18]. Another study in Norway (*n* = 56) found that PFHxS, PFNA, PFOA, and PFOS measured at delivery in mothers was associated with lower early childhood rubella antibodies among children (age three years) who received one dose of the MMR vaccine [48].

We were unable to compare our findings on MeFOSSA and EtFOSSA, due to a gap in evidence for how these specific PFAS may impact vaccine-induced immunity. To our knowledge, this is the first epidemiological study to examine prenatal MeFOSAA and EtFOSAA in relation to childhood vaccine antibody responses. While recent investigations have demonstrated associations between PFAS exposures and altered vaccine antibody levels in children, these studies have focused primarily on the legacy compounds PFOS, PFOA, PFNA, and PFHxS [14, 49]. MeFOSAA and EtFOSAA are intermediate compounds to PFOS, were phased out in the early 2000s, and are important for biomonitoring purposes as direct markers of historical exposure to PFOS precursors [50–52]. Our findings of distinct and divergent associations for these precursors suggest these compounds may have independent immunomodulatory effects warranting further investigation.

Animal models suggest that PFOA and PFOS alter innate immune responses through inflammatory processes and adaptive responses via alterations in cytokine production [53]. PFAS interfere with processing and presentation of antigens and modulate cell populations involved in antibody responses [1, 53, 54]. While limited toxicological data exist specifically for MeFOSAA and EtFOSAA, available evidence suggests these precursors may have distinct biological activities beyond their role as PFOS sources [55]. These differential molecular interactions may explain the divergent vaccine-specific associations we observed. We hypothesize that our findings may be due to alterations in prenatal fetal immune system programming, which is sensitive to exogenous exposures that could have long lasting effects [3]. However, the biological pathways by which PFAS impact fetal immune development and immunity in childhood are not understood. During the prenatal period, most of the innate immune system is established, largely from the transplacental transfer of pathogen-specific maternal IgG antibodies [56, 57]. Major fetal developmental events include hematopoiesis initiation, stem cell migration, progenitor cell expansion, and colonization of the marrow and thymus [3]. This period of fetal development is sensitive to exogenous exposures, some of which may have lasting effects on the developing immune system [3].

Furthermore, PFAS may induce toxicity via their interaction with the peroxisome proliferator-activated receptors (PPARs), which are expressed in numerous tissues, including the placenta, and are important in several molecular pathways, including the regulation of placental functions, cell proliferation, and inflammation, in a sexually dimorphic manner [58, 59]. We think it is possible that the molecular mechanisms by which prenatal PFAS may impact fetal immune system development with sex-specific differences could be partially explained by the intersection of PFAS, the placenta, and PPAR signaling pathways, although more studies are needed. In addition, evidence exists that PFAS exposure is associated with overall differential placental DNA methylation, as well as sex-specific placental epigenetic responses, linked to genes involved in many health outcomes, including immune functions, placental dysfunction, and pregnancy outcomes [60]. The vaccine-specific nature of our two main findings may also reflect the distinct immune pathways each vaccine engages, as measles (live attenuated viral) and pertussis (acellular protein) vaccines elicit different Th1 versus Th2 cytokine profiles [61].

Maternal blood PFAS might represent recent or long-term exposure, depending on half-life, usually years [62]. PFHxS has a half-life of about five years in serum, while PFOA and PFOS have half-lives around three years [63]. However, little is known about the kinetics of PFAS circulation in pregnancy, and circulating blood levels may have different trimester-specific implications on immune system development, especially due to rapid maternal physiological changes and different stages of placental development [64]. The majority of transplacental transfer of IgG to the fetus occurs in the last trimester of gestation, and only about 10% are thought to be transferred during the first trimester [65]. It is possible that trimester differences in immune system development could explain why we observed few associations between first trimester PFAS and childhood antibody titers, compared to studies examining second and third trimester PFAS.

Our study had several limitations. First, our exposure assessment did not capture the time-varying nature of PFAS exposure or allow for identification of susceptible periods of development. Second, we measured fewer antibody titers for rubella, pertussis, diphtheria, and tetanus which resulted in a loss of statistical power, especially for tetanus which had the fewest measurements. Third, we did not adjust our findings for multiple testing due to the relatedness of the associations (hypotheses are not mutually independent) and prior studies suggesting the global null is unlikely [66], and it is possible some findings could be due to chance.

Despite these limitations, we harnessed a relatively large analytical sample size of around 400 children and examined associations between PFAS and multiple antibody titers, including pertussis antibody titers which were not included in prior studies. We utilized objective blood PFAS measurements, which may be most predictive of what transports into fetal circulation during pregnancy. We adjusted for variables such as time since last MMR or DTaP vaccination and restricted the population to children who were fully vaccinated, strengthening the findings. We also examined prenatal PFAS in the first trimester, filling in an important literature gap, as the first trimester is likely reflective of PFAS concentration during the entire pregnancy due to long half-lives of the compounds [67], and most prior studies examined immune-mediated outcome associations with postnatal exposures.

## Conclusions

We found no associations between the first trimester PFAS mixture and antibody titers to measles, mumps, rubella, pertussis, diphtheria, and tetanus at age seven in this fully vaccinated population. We found a few inconsistent findings between individual PFAS and antibody titers. Overall, we found limited evidence of associations between first trimester plasma PFAS and mid-childhood antibody titers in our cohort, and our findings highlight the complexity of the impact of prenatal PFAS on the developing immune system. It will be valuable to continue elucidating trimester-specific impacts of PFAS and other exogenous exposures on immune system development.

## Supplementary Information

Supplementary Material 1.

## Data Availability

Because of the sensitive nature of the data collected for this study, requests to access the data set from qualified researchers trained in human subject confidentiality protocols may be sent to Project Viva at project_viva@hphci.harvard.edu and to the corresponding author. Data can be accessed with the appropriate permission from the Project Viva study team following the study policies (https://www.hms.harvard.edu/viva/).
