# Supplementary material for: Associations of prenatal exposure to per- and polyfluoroalkyl substances (PFAS) and childhood vaccine-induced immunity
Source: Environ Health. 2026 Jul 15;25:79. doi: 10.1186/s12940-026-01322-7 (PMC13647844; doi:10.1186/s12940-026-01322-7)
Supplement: Supplementary file 1 [file 12940_2026_1322_MOESM1_ESM.docx]

**Supplementary Information**

**Table S1.** Categorical distributions of antibody titers in mid-childhood blood among participants with prenatal PFAS measurements, at least one antibody titer outcome, and a complete MMR or DTaP vaccine series.

| Antibody Titers | Samples Tested (*n*) | Above seroprotective threshold^1^  *n* (%) | Borderline^1^  *n* (%) | Below seroprotective threshold^1^  *n* (%) |
| --- | --- | --- | --- | --- |
| Measles | 416 | 214 (51.4) | 92 (22.1) | 110 (26.4) |
| Mumps | 416 | 399 (95.9) | 6 (1.4) | 11 (2.6) |
| Rubella | 402 | 350 (87.1) | 24 (6.0) | 28 (7.0) |
| Pertussis | 396 | 6 (1.5) | 26 (6.6) | 364 (92.0) |
| Diphtheria | 388 | 388 (100) | 0 (0) | 0 (0) |
| Tetanus | 333 | 369 (100) | 0 (0) | 0 (0) |

Abbreviations: *n*, sample size; PFAS, per- and polyfluoroalkyl substances.

^1^Based on assay recommendations and not on universal guidelines.

**Table S2.** Sex-stratified associations of prenatal blood PFOS (ng/mL) and pertussis among females (*n*=191) and males (*n*=214).

| Females  Pertussis %Δ (95% confidence interval)^a^ | Males  Pertussis %Δ (95% confidence interval)^a^ |
| --- | --- |
| -0.9 (-2.0, 0.2) | 1.0 (-0.1, 2.1) |

Abbreviations: %Δ, percent change; PFOS, perfluorooctane sulfonate.

^a^We log-transformed the pertussis antibody titer outcomes and estimates and confidence intervals were converted using the formulas (e^β^-1)*100 and (e^95% CI upper or lower bound^-1)*100 and interpreted as the relative change in median pertussis antibody titer [(%Δ (95% CI)] when adding one unit of PFOS concentration. Models adjusted for assigned child sex at birth, time since last vaccination, and parity.

**2128** live births

**2100** maternal-child pairs included in present analyses

[kept first live birth only, for participants with more than one live birth]

**1627** participants with all maternal first trimester blood PFAS measurements

**523** children with at least one antibody titer outcome measured at mid-childhood

**486** children with medical record data

**424** children with 2 Doses MMR vaccine

**414** children with 5 Doses DTaP vaccine

**412** children with 5 Doses DTaP vaccine at recommended time

**423** children with 2 Doses MMR vaccine at recommended time

**405** children with 5 Doses DTaP vaccine at recommended time and more than 14-day difference between antibody titer blood draw and 2^nd^ vaccine dose

**416** children with 2 Doses MMR vaccine at recommended time and more than 14-day difference between antibody titer blood draw and 2^nd^ vaccine dose

**Figure S1.** Flow chart of participant inclusion.

Mid-childhood antibody titers

Prenatal PFAS (first trimester)

Vaccine status

Maternal education

Housing or work location (rural vs. urban) or healthcare access

Maternal age

Maternal race and ethnicity

Structural racism, redlining

Vaccine beliefs

Maternal religion or family values

Time since last vaccination

Parity

Child sex at birth

Breastfeeding & milk composition current pregnancy

**Figure S2.** Directed acyclic graphs (DAGs) for the exposure-response relationship between prenatal PFAS (first trimester) (green or without an outline) and mid-childhood antibody titers (orange or without an outline) to measles, mumps, rubella, pertussis, diphtheria, tetanus, in a fully vaccinated population. The blue or circled variables are those we adjusted for in final models (parity, time since last vaccination, child sex at birth). To account for the variable vaccine status (purple or without an outline), included in the DAG, we included only individuals fully vaccinated with the MMR and DTaP vaccine series in our analytical sample.

**B**

**A**

**Figure S3.** Spearman correlation coefficients among prenatal PFAS measurements in participants with at least one antibody titer outcome, and **(A)** a complete MMR vaccine series (*n*=416) or **(B)** a complete DTaP vaccine series (*n*=405).

**Figure S4.** Spearman correlation coefficients among continuous antibody titer outcomes in participants with at least one antibody titer outcome and a complete MMR and DTaP vaccine series (*n*=383).
